# Dynamically Tunable Friction via Subsurface Stiffness Modulation

**DOI:** 10.3389/frobt.2021.691789

**Published:** 2021-07-01

**Authors:** Siavash Sharifi, Caleb Rux, Nathaniel Sparling, Guangchao Wan, Amir Mohammadi Nasab, Arpith Siddaiah, Pradeep Menezes, Teng Zhang, Wanliang Shan

**Affiliations:** ^1^ Department of Mechanical and Aerospace Engineering, Syracuse University, Syracuse, NY, United States; ^2^ Department of Mechanical Engineering, University of Nevada, Reno, NV, United States; ^3^ Mechanical and Industrial Engineering Department, Montana State University, Bozeman, MT, United States

**Keywords:** dynamically tunable friction, subsurface stiffness modulation, low melting point alloy, soft robots, untethered crawling robots

## Abstract

Currently soft robots primarily rely on pneumatics and geometrical asymmetry to achieve locomotion, which limits their working range, versatility, and other untethered functionalities. In this paper, we introduce a novel approach to achieve locomotion for soft robots through dynamically tunable friction to address these challenges, which is achieved by subsurface stiffness modulation (SSM) of a stimuli-responsive component within composite structures. To demonstrate this, we design and fabricate an elastomeric pad made of polydimethylsiloxane (PDMS), which is embedded with a spiral channel filled with a low melting point alloy (LMPA). Once the LMPA strip is melted upon Joule heating, the compliance of the composite structure increases and the friction between the composite surface and the opposing surface increases. A series of experiments and finite element analysis (FEA) have been performed to characterize the frictional behavior of these composite pads and elucidate the underlying physics dominating the tunable friction. We also demonstrate that when these composite structures are properly integrated into soft crawling robots inspired by inchworms and earthworms, the differences in friction of the two ends of these robots through SSM can potentially be used to generate translational locomotion for untethered crawling robots.

## Introduction

Recently, the field of soft robotics has been growing rapidly and opening up possibility of achieving new maneuvers and locomotion approaches that cannot otherwise be accomplished by conventional hard robots (Laschi et al., 2016). Many of these soft robots are inspired by biological creatures and processes. Untethered soft robots can potentially match the abilities of these biological creatures. These soft robots have many potential applications including surveillance (Wu et al., 2008; Song et al., 2009; Lee et al., 2011), search-and-rescue missions (Kamegawa et al., 2004; Kamikawa et al., 2004; Wright et al., 2007), space exploration (Goldberg et al., 2002; Fong and Nourbakhsh, 2005), and others (Yap et al., 2015a, Yap et al., 2015b; Cianchetti et al., 2015; Di Luca et al., 2017).

In nature, many soft crawling animals make movements by shortening and lengthening their bodies (Li et al., 2011; Kovač, 2014; Calisti et al., 2017; Zimmerman and Abdelkefi, 2017). Many studies focused on mimicking these shortening/lengthening maneuvers to achieve robotic locomotion (Trimmer et al., 2006; Umedachi et al., 2013). For example, Trimmer et al. developed a caterpillar robot using shape memory alloy (SMA) springs and elastomers, which is able to deform and crumple into a small volume (Trimmer et al., 2006). In another study a 3D-printed soft robot was introduced, which is able to generate inching and crawling locomotion (Umedachi et al., 2016). This soft robot’s feet are made of two different materials with different coefficients of friction (CoF). The posture of the robot can be changed to change the CoF of the robot bases. Most recently, Huang et al. introduced a bioinspired soft robot with actuation from SMA wires. To overcome the longstanding issue of long cooling time for SMA actuators, the SMA wires were embedded in a thermally conductive elastomer (Huang et al., 2019).

Many of the soft crawling robots that have been developed rely on pneumatics and asymmetry in structure and geometry for locomotion (Jung et al., 2007; Godage et al., 2012; Zhou et al., 2017; Ge et al., 2018). Shepherd et al. developed a pneumatically actuated multi-gait soft crawling robot, which is able to do sophisticated locomotion, including crawling and undulating underneath a short gap (Shepherd et al., 2011). This design of soft crawling robot relies on pneumatics combined with asymmetry in the geometry and structure of the robot body to generate locomotion. In a more recent work by Tang et al., a switchable adhesion actuator was introduced for the gripping feet of a crawling robot (Tang et al., 2018). Adhesion switching is achieved by applying positive pneumatic pressure into embedded spiral channels in an elastomer plate, which also contains a cylindrical chamber underneath the channels. When pressurized, the spiral channels expand and the cylindrical chamber’s volume increases, which creates adhesion of the whole elastomer plate to the adhering substrates. In another study, the potential design of 1D soft crawling robots based on dynamically tunable friction coefficient is explored using the combination of theory and simulation (Zhu et al., 2017).

**FIGURE 1:**
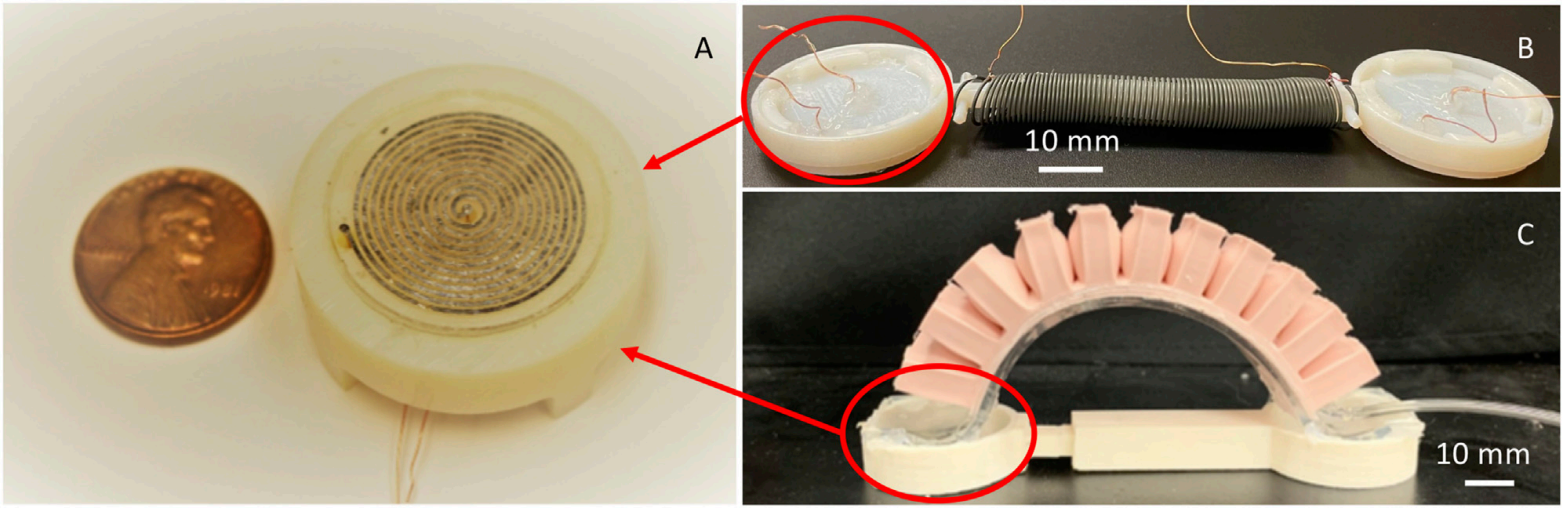
**(A)** Close-up view of the composite pad with tunable CoF **(B)** The soft crawling inchworm inspired robot with two composite pads at the ends. **(C)** The soft crawling earthworm inspired robot with two composite pads at the ends.

In this work, we explore a novel approach to dynamically tunable friction through subsurface stiffness modulation (SSM) (Figure 1), inspired by recent work on dynamically tunable adhesion through SSM for robotic manipulation (Tatari et al., 2018). Here, we first develop a robust fabrication method for composite pads containing subsurface components with tunable stiffness, then we characterize the dynamically tunable frictional behavior of the composite pads using a tribometer. Finite Element Analysis (FEA) is employed to qualitatively identify the mechanism that contributes to the observed tunable friction. The effect of certain design parameters including the sealing layer thickness on tunable friction for the composite pads is also explored. Toward the end, we demonstrate the application of these composite pads in two soft crawling robots inspired by earthworms and inchworms. The movements of these soft crawling robots are powered by either a two-way nitinol SMA spring (Figure 1B) or pneumatics (Figure 1C). The dynamically tunable friction approach introduced here, combined with non-pneumatic activation mechanisms, can potentially enable many applications in untethered versatile soft crawling robots.

## Materials and Methods

**FIGURE 2:**
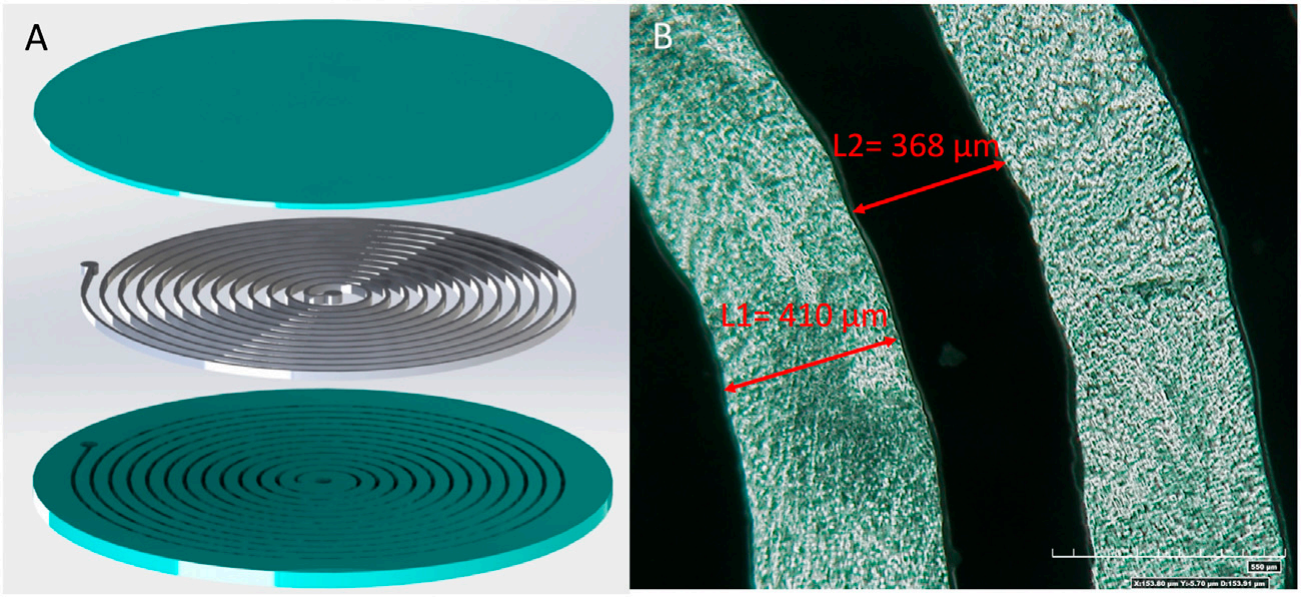
**(A)** Exploded view of the schematics for composite pads with tunable CoF, PDMS (Blue) LMPA (Silver). **(B)** Optical image of the fabricated LMPA channels (shiny) in the composite pads **(top view)**.

Friction between surfaces depends on a suite of mechanical and geometrical parameters including surface roughness and mechanical properties of the substrates. Here we propose to dynamically change the frictional behavior of an elastomeric pad by changing the stiffness of embedded channels filled with phase-changing materials. Figure 2A shows the schematics of the exploded view of this design. Polydimethylsiloxane (PDMS), a commonly used material for soft robotics, is chosen as the material for the elastomeric bulk. Low melting point alloys (LMPAs), which allow for both fast phase change and large stiffness change, are chosen as the stimuli-responsive material. A composite pad with a circular shape is fabricated by embedding spiral channels filled with an LMPA, Roto 144F Low Melt Fusible Ingot Alloy (or Field’s metal) (RotoMetals, Inc.) These channels are positioned 350–750 μm away from the contact/working interface. The channels have been designed to have a rectangular cross section with a width of 300 μm and a height of 500 μm. The LMPA channels can be activated by running an electric current of approximately 1 A for a short period of seconds.

Through Joule heating, the LMPA channels absorb enough heat to transform from a solid phase to a liquid phase. Due to this phase change, the rigidity of the channels decreases to zero, and they have the ability to return to their solid state after deactivation, which is the process where no electric current is supplied to the electrodes of the PDMS-LMPA composite pads. During deactivation, the LMPA channels become solid once again due to heat dissipation into the surroundings. It is expected that by tuning the rigidity of the LMPA channels, which are embedded close to the surface of the PDMS-LMPA composite pad, the CoF of the composite pad is also tuned.

It should be mentioned that the final design of the composite pads has been achieved through a trial-and-error procedure. The geometry of the channels including the thickness, the width, the length, and the spacing between the channels have been changed to obtain higher changing ratio of the CoF before and after activation. For example, by increasing the thickness and the width of the spiral channels while keeping the channel spacing constant, we will have a more rigid composite pad due to the increasing volume percentage of the LMPA material. However, there is a limitation on how much these dimensions can be increased, as by making the channels thicker and wider, the electrical resistance of the spiral channels is much reduced, which makes it harder to activate the sample. Therefore, there is a tradeoff between increasing the stiffness and decreasing the electrical resistance of the composite pad. The final design presented here is achieved based on the trial-and-error experimental results. However, it certainly is still not the best design yet in terms of achieving higher friction tunability. To find the globally optimal design, serious optimization effort is needed, which is beyond the scope of this work.

### Fabrication

**FIGURE 3:**
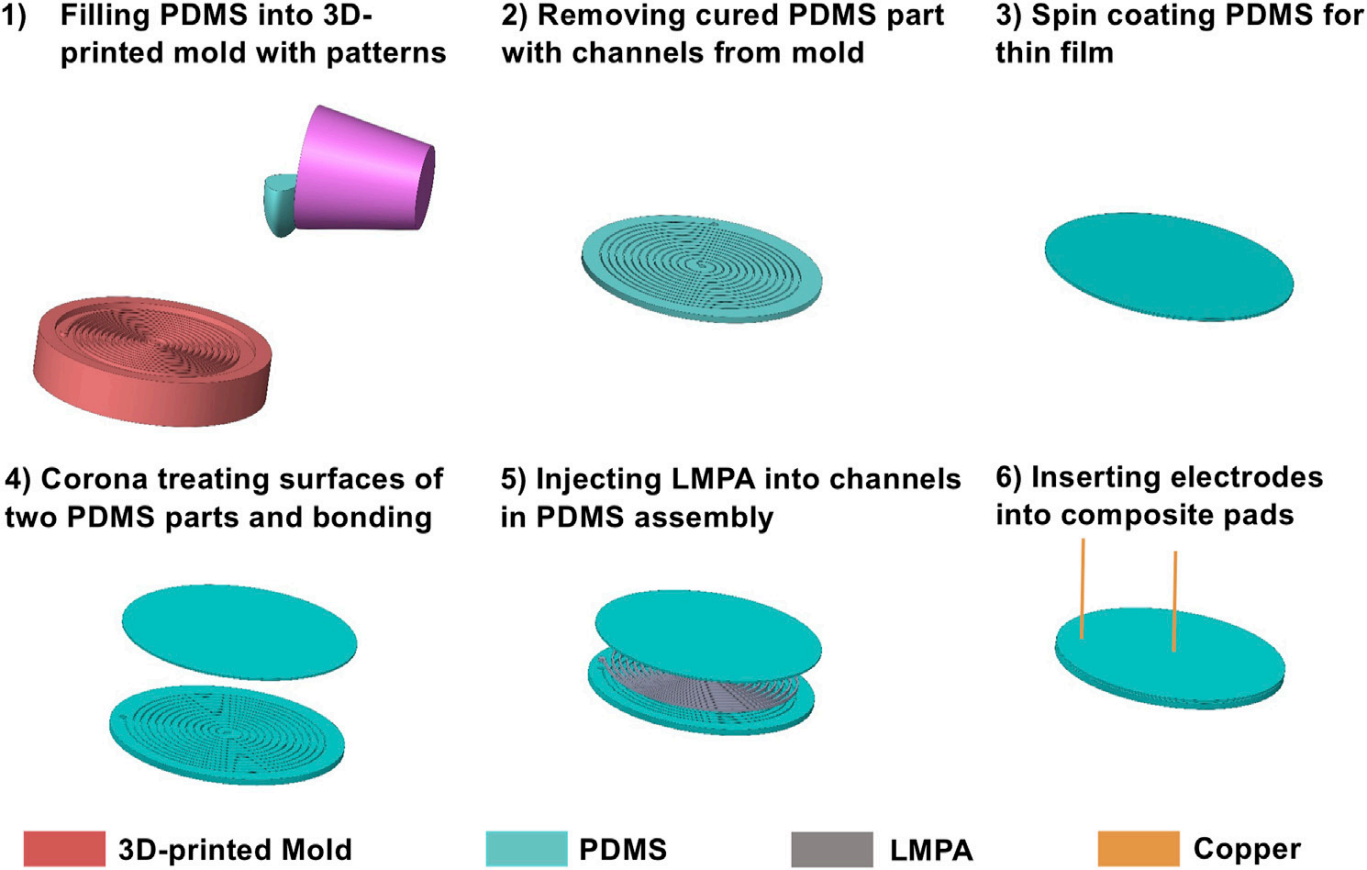
Schematics of the fabrication process.

The composite pads are fabricated through a multi-step process, which is shown in Figure 3 schematically. First, uncured PDMS is cast into a 3D printed mold to have the bottom part as shown in Figure 2A, which contains the channels exposed on the top side (Step 2). Then, a thin layer of PDMS is made by spin coating for sealing the channels from above (Step 3). After obtaining the bottom PDMS part and the top PDMS sealing layer, they are bonded together after the surfaces intended to be in contact are treated with a plasma gun (Step 4). At this stage, a circular PDMS plate containing empty spiral channels is obtained. We then vacuum-filled liquid LMPA into the channels (the middle part in Figure 2A) following a procedure described in Ref. (Lin et al., 2017). and put two copper wires at the two ends (wells) as the electrodes (Step 5 and 6). The fabricated composite pad-like structure with tunable friction is shown in Figure 1B.

In practice, due to the resolution of the 3D printing, the cross section of the channels is not exactly rectangular, with the top width bigger than 300 μm **(** Figure 2B). Nonetheless, the average width of the channels is close to the designed value. Before experimental characterization of these composite pads, they are attached to 3D-printed fixtures using silicone adhesives. This assembly is further examined to ensure that the embedded circuit achieves continuity before experimental characterization of their frictional behavior.

### Experiments

In order to measure the CoF of the composite pads, a multifunctional tribometer (Rtec MFT 5000) has been used. In the experiments, a ball of either steel or ceramic alumina is dragged across the sample’s surface with a constant speed of 2 mm/s and a normal force of 2 N. The dragging speed of 2 mm/s is used to achieve sliding conditions under boundary lubricated regime where asperity-asperity (surface-surface) contact dominates (Menezes et al., 2011, Menezes et al., 2013; Menezes and Kailas, 2016). The 2 N normal force is small enough such that the steel/ceramic ball does not damage the soft composite pads during sliding, while at the same time large enough such that the data is not buried in noise.

**FIGURE 4:**
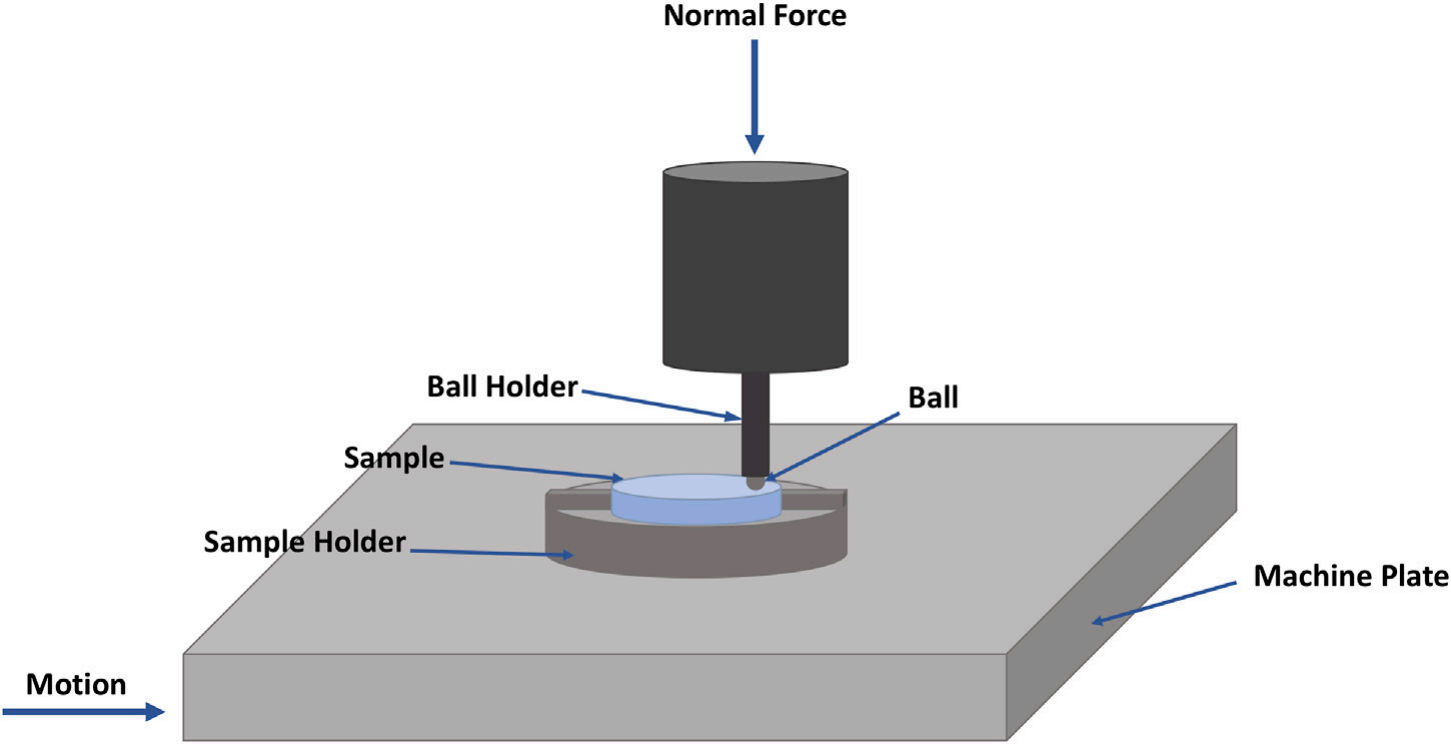
Schematics of the friction characterization setup.

Data for the CoF between the ball and the composite pad sample is acquired during the entire sliding distance by the testing system of the tribometer. A sample holder was 3D-printed for fastening the composite pad sample to the test bed. The rigid encasing that holds the sample in place during the sliding of the ball is also illustrated in Figure 2A (the white segments on the circumference of the three parts). In Figure 4, a schematic detailing the tribometer setup, including the sliding direction, is shown.

**FIGURE 5:**
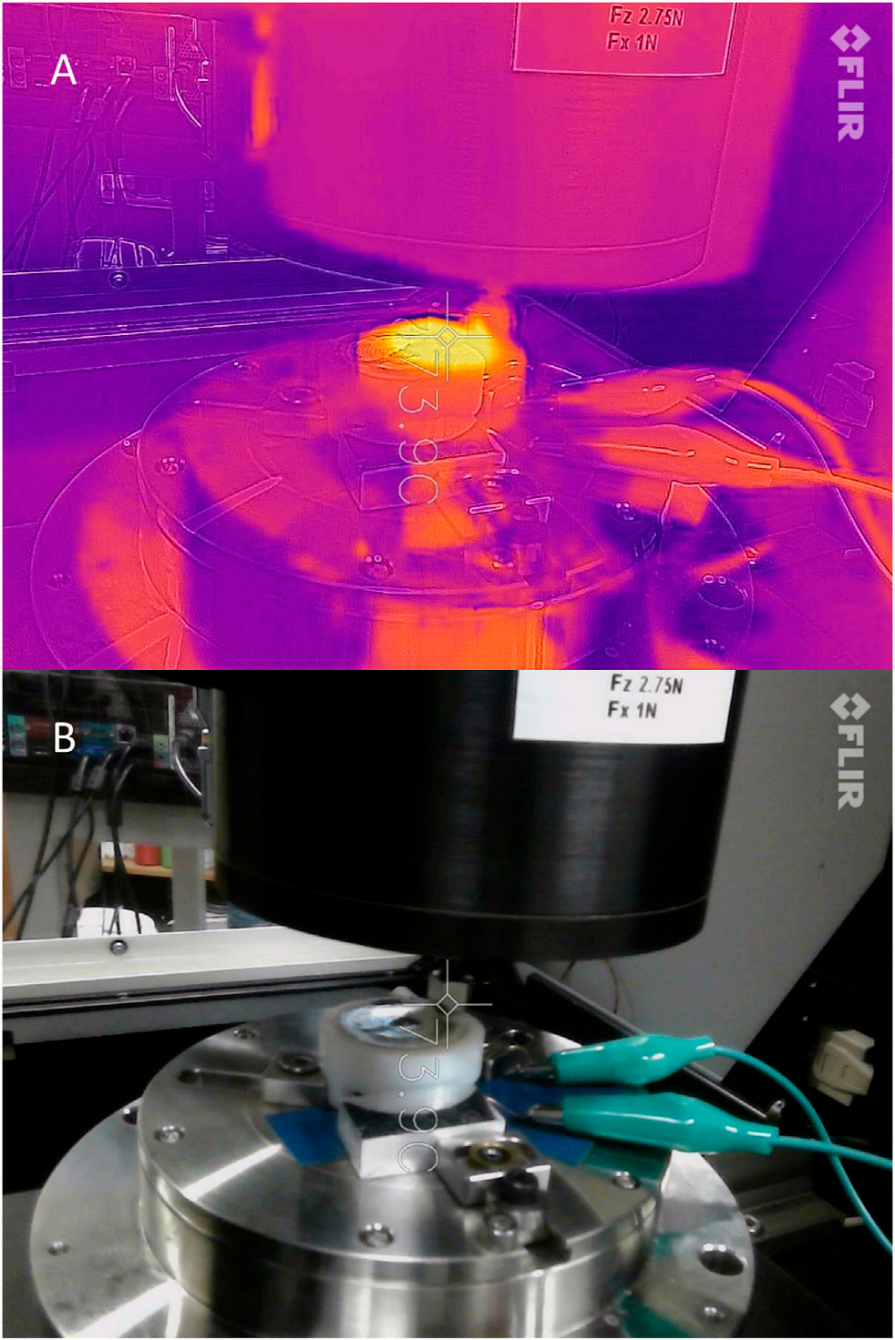
Snapshots captured by a thermal camera to measure the surface temperature of the composite pad during the experiments. **(A)** Thermal view. **(B)** Normal view.

Each composite pad was secured in the tribometer while single-line scratch tests were performed with a steel ball. Three tests were completed while the composite pad was in its non-activated state at room temperature without an applied voltage. Following that, more tests were performed once the pads were properly activated using Joule heating. That is, when the surface temperature reached between 70–75°C, approximately 10 °C above the melting point (62°C) of the LMPA, Field’s metal. A thermal camera (FLIR ONE Pro LT) is used to monitor the temperature of the surface of the composite pads during the experiment in real-time. A snapshot obtained by the thermal camera during experiment is illustrated in Figure 5. This snapshot also contains an optical image of a composite pad sample during the test. Supplementary Video S1 demonstrates the activation and deactivation process of the composite pads in lab air using a constant input voltage of 2 V and a current of ∼1 A. After the tests, the CoF data is collected, processed, and analyzed by MATLAB software.

### Modeling

**FIGURE 6:**
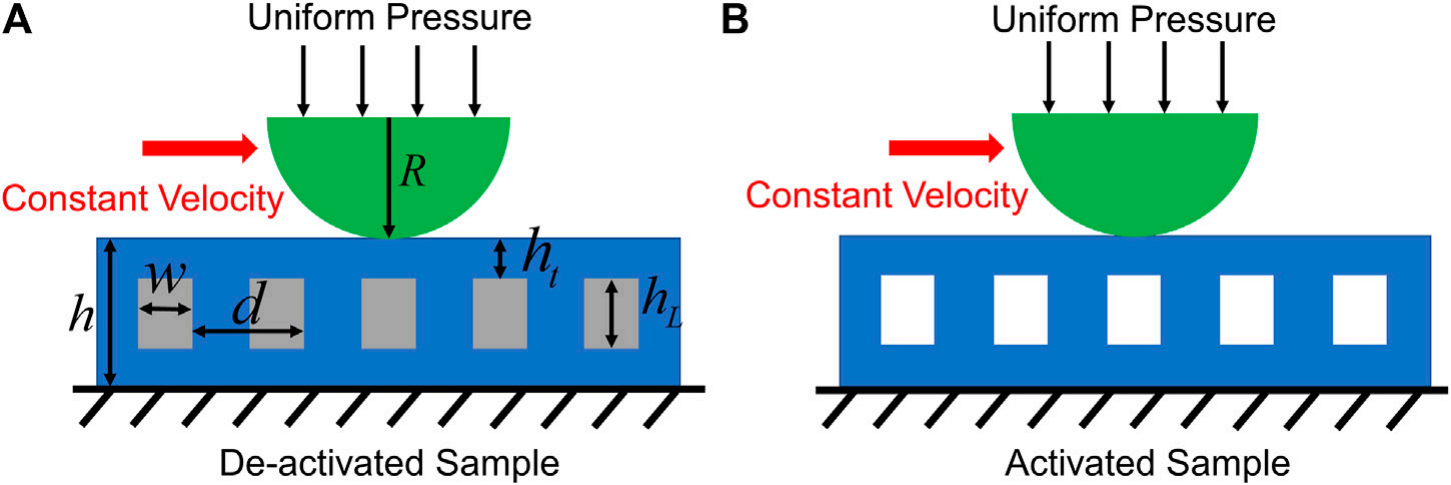
Schematic illustration of the geometry model in ABAQUS for deactivated **(A)** and activated **(B)** composite pad sample. The geometric parameters are *R* = 1.585 mm, *h* = 1.2 mm, *w* = 0.2 mm, *d* = 0.8 mm, $h_{L}$ = 0.5 mm, and $h_{t}$ = 0.4 mm.

To understand the underlying physics that governs the tunable friction of the composite pads, we resort to FEA based on the commercial software ABAQUS to qualitatively study the friction of the composite samples against the steel ball used in tribology experiments. For simplicity without losing the essence, we reduce the problem to a 2D plane strain one to mimic the symmetric plane of the pad disk, and the geometry of the model is shown in Figure 6. The same geometry and material properties as those in experiments are used throughout the simulations (Table 1). It is worth mentioning that when the LMPA is activated, we replace the rigid components with voids since the modulus of the liquid LMPA is so small that we can ignore its effect on the composite pad’s deformation.

**TABLE 1:** The material properties of the composite pad sample that are used in the FEA simulation (Mohammadi Nasab et al., 2020).

	Young’s modulus (MPa)	Density (kg/m^3^)	Poisson’s ratio
PDMS	2.1	965	0.475
Steel	210,000	7,850	0.3
LMPA (activated)	∼0	9,700	0.5
LMPA (deactivated)	9,250	9,700	0.3

The simulation consists of two steps as in the tribology tests. In the first step, a general static analysis is performed in which the indenter is pushed against the composite pad through a uniformly distributed pressure. The nonlinear geometry option is turned on, and the default automatic stabilization scheme is used to help convergence (the dissipated energy fraction is specified as 0.0002 by default). In the second step, dynamic implicit analysis is used when the indenter moves horizontally with a constant velocity while the normal force is kept constant (Sun et al., 2019). The quasi-static application is chosen for this step. During the entire process, the bottom surface of the composite pad is fixed, which corresponds to the boundary conditions in experiments. It should be noted that the total normal force that is applied in simulations is smaller than that in experiments because the simulations are based on a 2D model, which is different from the experiments. In fact, it is found that using a 2 N normal force will create significant distortion of the composite pad when the LMPA is activated and thus lead to convergence problem.

The indenter and the pad are divided into 8-node biquadratic plane strain elements with reduced integration (CPE8R), and a mesh refinement study is carried out to make sure the convergences of the simulations. Surface-to-surface interaction is set up between the top surface of the composite pad and the indenter. Besides, contact between the sidewalls of the LMPA channels is also considered when the LMPA is activated. For the interaction settings, the normal behavior is set up as “hard” contact while the tangential behavior is set up as penalty friction. The friction coefficient is chosen as 0.5 and the shear stress limit is chosen as 0.3 MPa. Shear stress limit is observed in the interface between two surfaces (Sahli et al., 2018). However, we do not have experimental data to extract its value here. Therefore, the shear stress limit is changed in simulations to examine its effects. Simulations suggest that changing the shear stress limit will not affect the frictional behaviors in a qualitative way (Supplementary Material Presentation 1). In addition, increasing the shear stress limit beyond 0.5 MPa will lead to severe mesh distortion at the contact interface and make the results unrealistic. Therefore, these cases are not studied.

## Results and Discussion

**FIGURE 7:**
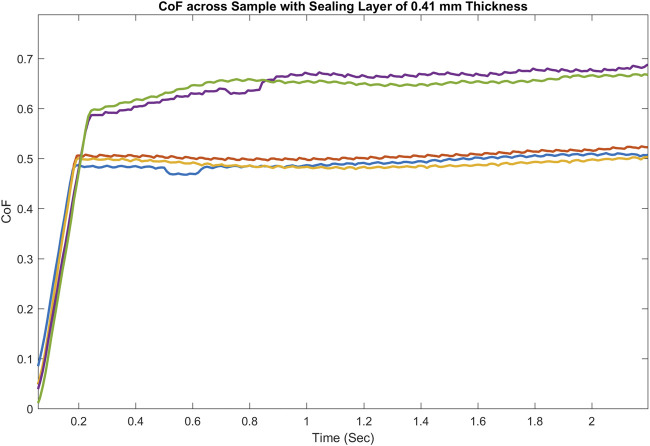
Example CoF vs. Time for the non-activated and activated composite pad samples.

In experiments, the CoF for the activated and non-activated cases of many composite pad samples with different thickness (between 350 and 750 μm) of the upper sealing layer (the top part in Figure 2A) has been tested. Figure 7 shows the plot of CoF vs. time for a sample with 420 μm thickness of the upper sealing layer, which has been tested with a steel ball. As shown, there is a considerable enhancement of CoF in the activated cases when compared to the non-activated ones.

**FIGURE 8:**
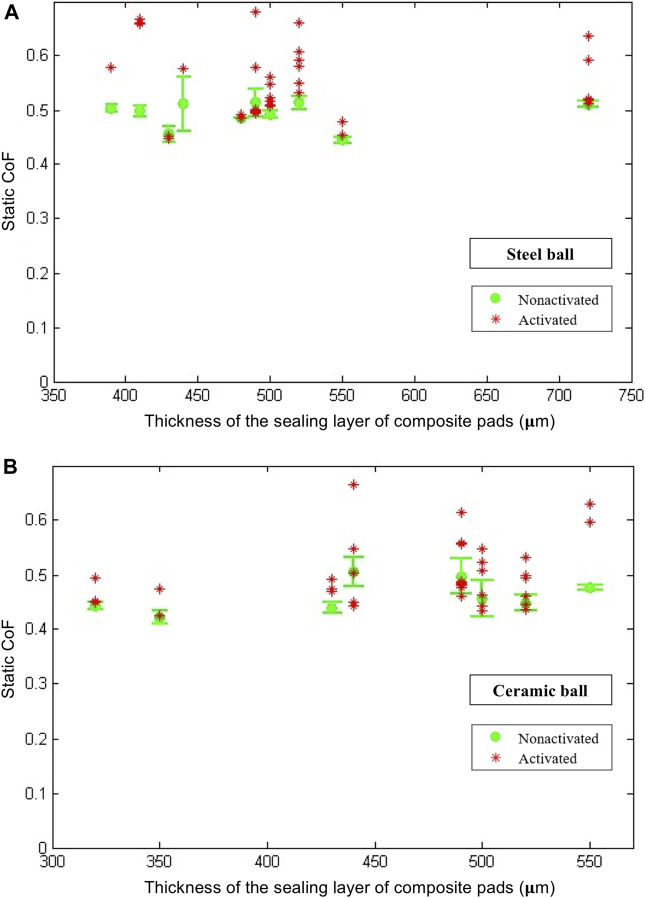
Static CoF between samples with different upper layer thicknesses and the steel/ceramic ball. At least three samples are tested for each of the nonactivated cases.

Also, the static CoF values for different upper sealing layer thicknesses, in both activated and non-activated cases, conducted with both a steel ball and a ceramic ball, have been illustrated in Figure 8. These plots show that the CoFs in the activated state are in general higher than their values in the non-activated case, despite the fact that the enhancement ratio varies in each sample. Such an enhancement is observed regardless of whether a steel ball or a ceramic ball is used for the testing. In certain cases, the CoF can be enhanced by up to 32%. Interestingly, this trend of increased friction when the subsurface component softens is quite the opposite to the trend of dynamically tunable adhesion through SSM, for which the dry adhesion is much lower when the subsurface component softens (Tatari et al., 2018).

Note that in the activated cases presented in Figure 8, for some samples, there are only one or two scratch test data points. The reason for this is, some of the composite pad samples failed after one or two tests due to the nature of the standard scratch test. Thus, for the activated cases, all successful data points are reported instead of an average with a standard deviation. Note also that, in many real applications during sliding, the local deformation on the surface of the composite pad would be much lower, and thus catastrophic failure of the composite pad would be much less frequent. Another point worth mentioning is that we used LMPA channels for activation, which is convenient but not reliable as the circuitry might break during loading when LMPA is in the solid phase. While reheating and resolidifying of the LMPA channels can restore the circuitry, novel smart materials such as the three-component ones containing LMPA inclusions (Mohammadi Nasab et al., 2020) will significantly improve the reliability of this approach to dynamically tunable friction. In addition, adopting these novel smart materials will also enable minimization and much quicker dynamic modulation of friction.

**FIGURE 9:**
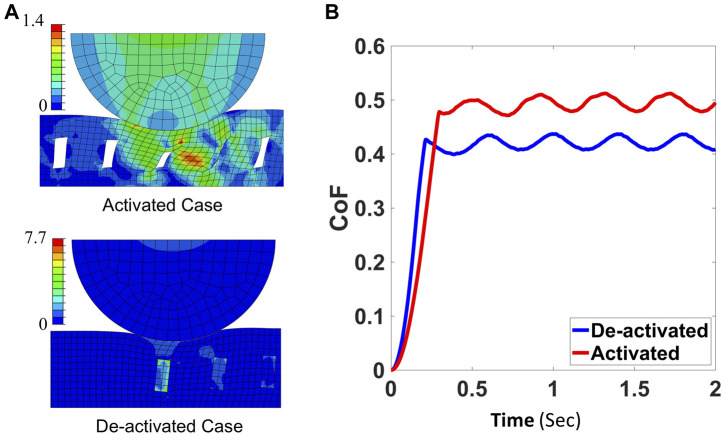
**(A)** The deformation of the deactivated and activated pads under normal force 0.951 N. The color represents the Mises stress (MPa). **(B)** The CoFs of the deactivated and activated composite pads from simulations.

We postulate that the enhanced friction when the sample is activated comes from the contact between the sidewalls of the embedded LMPA channels, and the evidence comes from the simulations as shown in Figure 9A. Here, the normal force is set as 0.951 N and the maximum shear stress that the interface can bear is set as 0.3 MPa. When the LMPA is deactivated and rigid, the channels barely deform during the sliding of the indenter due to the high Young’s modulus of the LMPA compared to the surrounding PDMS. However, when the LMPA melts and becomes liquid, the walls of the channels will contact themselves because of the large deformation caused by the shearing force of the indenter. As a result, the CoF will increase as demonstrated by the simulation results in Figure 9B.

The mechanism for the tunable friction that we discover here, in which the contact between the sidewalls of the embedded channels contributes to the increased surface friction, is consistent with the previous reports (He et al., 2016). However, we acknowledge that the answer is far from being definite. Many factors such as thermal expansion, viscoelasticity, and geometry can have an impact on friction, yet they have not been considered in our simulations. Among these, the thermal expansion effect is estimated to be small based on ∼50°C increase in the LMPA strips and the surrounding PDMS matrix. Nonetheless, further investigations are still needed to fully understand the underlying physics of the observed change of friction and quantify the contribution of each potential factor, which is beyond the scope of this work.

## Robot Design and Demonstration

To demonstrate the potential applications of these composite pads with dynamically tunable friction, two proof-of-concept model soft crawling robots have been developed: an inchworm-inspired soft robot, and an earthworm-inspired soft robot.

### Inchworm Inspired Soft Robot

#### Fabrication

An inchworm-inspired soft robot was designed with inspiration from other established soft robots. The inchworm robot was controlled through a fast-actuating pneumatic network containing 11 independent pneumatic nets (Mosadegh et al., 2014). These nets were designed to allow for greater actuation with less pressure, to increase the fatigue life of the robot.

The body of this soft robot is composed of three different components: a top piece that houses the pneumatic nets, a bottom piece that encloses the worm body, and a slider piece used for attaching the pads. To fabricate the top component, a two-piece mold was designed and 3D-printed. In addition, a mold for the bottom piece was designed and printed using the same practices described in *Fabrication*.

**FIGURE 10:**
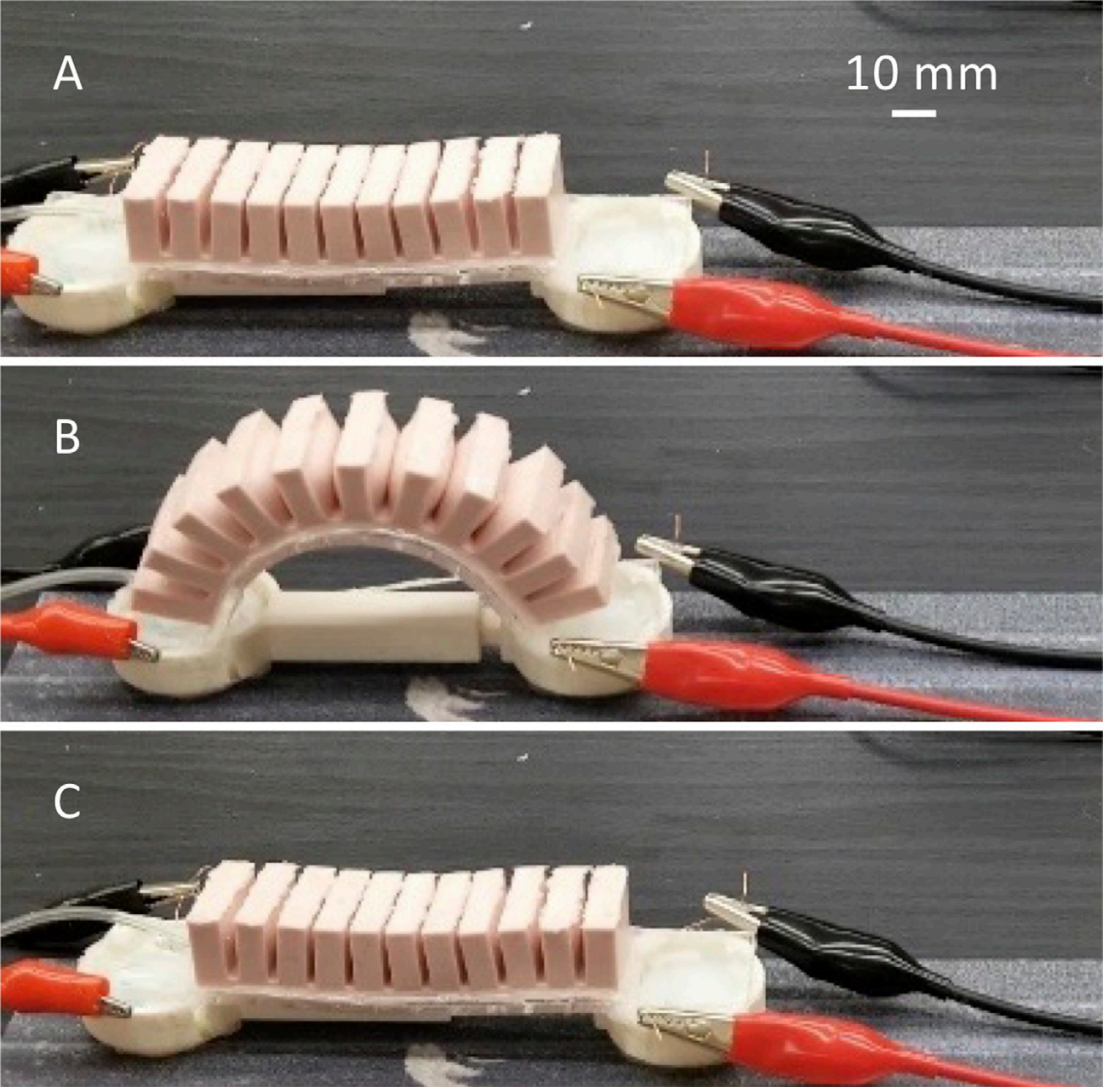
Illustration of the inchworm inspired soft robot crawling forward. **(A)** Activating the front pad. **(B)** By pressurizing the soft body, the rear pad moves forward. **(C)** Deactivating the front pad and activating the rear pad followed by releasing the air from the robot, the front pad moves forward.

The top pneumatic mesh is made of ELASTOSIL M4601. The bottom is composed of PDMS mixed at a 3:1 ratio rather than the recommended 10:1 ratio to create a stiffer component. The two were attached by adding a thin layer of PDMS and curing it in an oven. Due to the difference in stiffness between the ELASTOSIL M4601 and PDMS, the robot can curl into an inchworm shape when actuated (Figure 10). A 3D-printed slider was developed to connect the body of the robot to the pads, and to ensure that the pads are always in full contact with the substrate that the robot is crawling on (Tang et al., 2018). The robot was then glued at each end of the slider with Smooth-On Sil Poxy. The final assembled inchworm soft crawling robot is shown both in Figures 1, 10.

To actuate the inchworm soft robot, compressed air is supplied to the body through a surgical tube. The surgical tube is inserted at one end of the body and glued in place with Smooth-On Sil Poxy to ensure no air leaks. When effectively changing the friction between one end of the robot and the other, it could move forward similar to inch-worm locomotion, by anchoring one side and sliding the other forward. This could be done repeatedly to create net forward movement, as well as backward movement if the roles of the two ends flip.

#### Mechanism

In order to make the inchworm soft robot moving forward using the introduced composite pads, the composite pads need to be activated and deactivated in a sequence that is shown in Figure 10. First, the front pad is activated as in Figure 10A, then it should have higher friction than the rear pad. So, when the robot body is fully activated and bent, the front pad is effectively the anchor point and the rear pad moves forward, as shown in Figure 10B. This is followed by deactivation of the front pad and activation of the rear pad. Now the rear pad has higher friction than the front pad. Therefore, when the air is released from the inchworm soft robot’s chambers in Figure 10C, the rear pad is effectively the anchor point and the forward pad slides forward during straightening of the soft body. Effectively from sequences illustrated in Figures 10A–C, the inchworm soft robot crawls forward. This sequence of activation and deactivation can be repeated and modified to move forward and backward as needed. Supplementary Video S2 demonstrates how the inchworm robot moves forward using the procedures described here.

### Earthworm-Inspired Soft Robot

**FIGURE 11:**
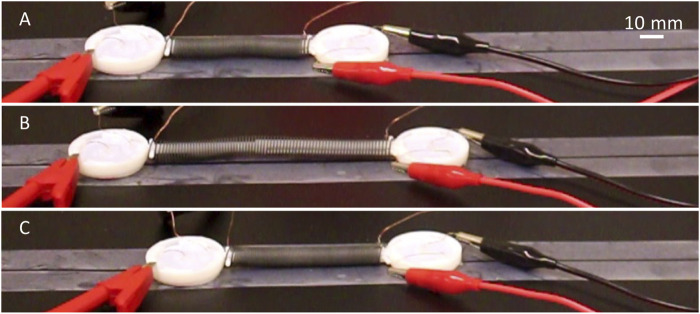
Illustration of the earthworm inspired soft robot crawling forward. **(A)** Activating the rear pad. **(B)** By extending the nitinol spring, the front pad moves forward. **(C)** Deactivating the rear pad and activating the front pad followed by deactivation of the nitinol spring the rear pad slides forward during contraction of the spring.

This earthworm soft robot is composed of three different parts: two composite pads, a two-way nitinol SMA spring, and a slider piece used for attaching the composite pads and the nitinol spring (Figure 1). The nitinol spring extends when activated (heated above 60°C) and contracts when deactivated (cooled down to room temperature or below), which can be used as the actuation mechanism. The 3D-printed slider connects the spring to the pads and ensures that the pads are always in full contact with the surface that the robot is crawling on. Two copper wires are attached to the nitinol spring as electrodes. The assembled earthworm-inspired soft robot is shown both in Figures 1, 11.

When the friction of the two ends of the soft robot against the surface it is climbing on is different, it can move forward by anchoring one end and sliding the other. This can be done repeatedly to create forward movement, as well as backward movement if the roles of the two ends flip. In order to make this robot move forward using the composite pads introduced earlier, the composite pads need to be activated and deactivated in a sequence that is shown in Figure 11. First, the rear pad is activated (Figure 11A), and it should have higher friction than the front pad. So, when the nitinol spring is activated, the rear pad is effectively the anchor point and the front pad moves forward, as shown in Figure 11B. This is followed by deactivation of the rear pad and activation of the front pad. Now the front pad has higher friction than the rear pad. Therefore, the front pad is effectively the anchor point and the rear pad slides forward during contraction of the spring (when the nitinol spring is deactivated) (Figure 11C). Effectively from sequences illustrated in Figures 11A–C, the soft robot crawls forward. This sequence of activation and deactivation can be repeated and modified to move forward and backward as needed. Supplementary Video S3 demonstrates that the earthworm inspired soft robot moves forward using procedures described here.

Note that here for both soft crawling robots the applied load on the composite pads is merely their self-weight, which is distributed across the whole contact surface, unlike in the tribology experiments and FEA simulations presented earlier. Nonetheless, these demos validated the general concept of dynamically tunable friction via SSM. Note also that in these specific implementations of soft crawling robots rigid 3D-printed sliders are incorporated for convenience, but our emphasis is on realizing dynamically tunable friction mechanisms that, if improved and optimized, can potentially be adopted ubiquitously in soft robotics. Last but not least, we have only explored the 1D crawling capability here, but the advantage of this SSM approach to dynamically tunable friction explored here lies in the cases where multiple composite pads are incorporated into one robot to explore 2D and 3D spaces. The use of electrically tunable CoF and locomotion directions will simplify the soft robot design and control significantly.

## Conclusion

In this paper, the concept of dynamically tunable friction through subsurface stiffness modulation has been introduced and validated with a composite pad structure containing subsurface low melting point alloy channels. This study presents a composite pad design with dynamically tunable friction, and a reliable fabrication method for these composite pads. Experimental characterization of the coefficient of friction of the composite pads structure has also been conducted, and finite element analysis has been used to understand the underlying mechanism for dynamically tunable friction observed in experiments. The results show that up to 32% enhancement in the CoF in the activated case can be achieved when compared with the nonactivated cases. It is also demonstrated that this dynamically tunable friction mechanism can be used to assist locomotion for soft crawling robots inspired by earthworms and inchworms, with potential to enable untethered soft crawling robots.

## Data Availability

The raw data supporting the conclusions of this article will be made available by the authors, without undue reservation.

## Supplementary Material

Supplementary Figure 1 The CoFs of the de-activated and activated composite pads from simulations. The normal force is kept as 0.951 N while the shear stress limit is 0.3 MPa for panel A and 0.4 MPa for panel B.

Supplementary Video 1 This video demonstrates the activation and deactivation process of the composite pads in lab air using a Keysight power supply. The input voltage was set as 2 V while the current (∼ 1 A) changed as the resistance of the LMPA channels increased with the increasing temperature.

Supplementary Video 2 This video demonstrates how the inchworm robot moves forward using the procedures illustrated in Figure 10.

Supplementary Video 3 This video demonstrates how the earthworm robot moves forward using the procedures illustrated in Figure 11.
