# Supplementary material for: Dynamically Tunable Friction via Subsurface Stiffness Modulation
Source: Front Robot AI. 2021 Jul 1;8:691789. doi: 10.3389/frobt.2021.691789 (PMC8281354; doi:10.3389/frobt.2021.691789)
Supplement: Supplementary file 2 [file Presentation1.pdf]

Supplementary Material

Dynamically Tunable Friction via Subsurface Stiffness Modulation

Siavash Sharifi, Caleb Rux, Nathaniel Sparling, Guangchao Wan, Amir Mohammadi Nasab, Arpith Siddaiah, Pradeep Menezes, Teng Zhang, Wanliang Shan

1 Finite Element Analysis (FEA)

This section provides details of the finite element simulations that are not provided in the main text. The simulations use the same geometry and material properties as in the experiments. The unit system that is adopted in ABAQUS is shown in **Table S1**.

Quantity	Length	Force	Mass	Time	Energy	Acceleration	Density	Stress
Unit	mm	N	g	ms	mJ	10^3 m/s^2	10^6 kg/m^3	MPa

Table S1. Unit system used in ABAQUS.

Figure S1 shows the CoFs of the activated and deactivated composite pad when the shear stress limit increases from 0.3 MPa to 0.4 MPa. The other parameters are kept fixed. It turns out that the activated composite pad still has higher CoF than that of the non-activated pad, indicating that the change of the shear stress limit will not alter the frictional behaviors qualitatively.

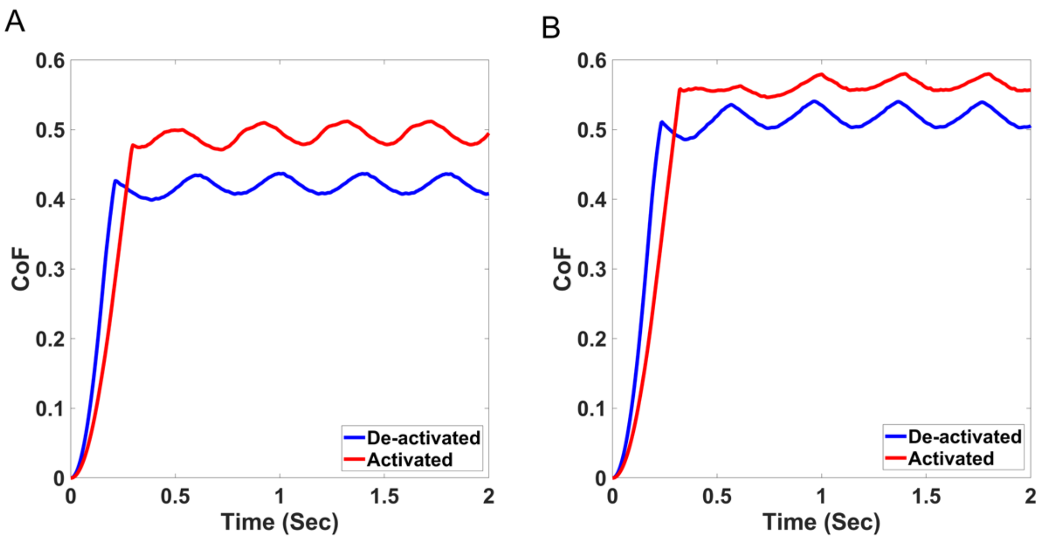

Figure S1. The CoFs of the de-activated and activated composite pads from simulations. The normal force is kept as 0.951 N while the shear stress limit is 0.3 MPa for panel **A** and 0.4 MPa for panel **B**.

2 Supplementary Videos

Video S1. This video demonstrates the activation and deactivation process of the composite pads in lab air using a Keysight power supply. The input voltage was set as 2 V while the current (~ 1 A) changed as the resistance of the LMPA channels increased with the increasing temperature.

Video S2. This video demonstrates how the inchworm robot moves forward using the procedures illustrated in **Figure 10**.

Video S3. This video demonstrates how the earthworm robot moves forward using the procedures illustrated in **Figure 11**.
